# Assessment of Changes in Cap and Residual Stromal Thickness Values during a 6-Month Observation after Refractive Lenticule Extraction Small Incision Lenticule Extraction

**DOI:** 10.3390/jcm13072148

**Published:** 2024-04-08

**Authors:** Dominika Janiszewska-Bil, Barbara Czarnota-Nowakowska, Izabela Kuciel-Polczak, Dariusz Dobrowolski, Beniamin Oskar Grabarek, Anita Lyssek-Boroń, Edward Wylęgała, Joanna Wierzbowska

**Affiliations:** 1 Department of Ophthalmology, Trauma Centre, St. Barbara Hospital, 41-200 Sosnowiec, Poland; i.kucielpolczak@gmail.com (I.K.-P.); dardobmd@wp.pl (D.D.); anitaboron3@gmail.com (A.L.-B.); 2 Optegra Clinic in Katowice, 40-101 Katowice, Poland; 3 Collegium Medicum, WSB University, 41-300 Dabrowa Gornicza, Poland; bgrabarek7@gmail.com; 4 Optegra Clinic in Poznań, 61-101 Poznań, Poland; barbara.czarnota@icloud.com; 5 Clinical Department of Ophthalmology, Faculty of Medical Sciences in Zabrze, Medical University of Silesia, 40-760 Katowice, Poland; wylegala@gmail.com; 6 Department of Ophthalmology, District Railway Hospital, 40-760 Katowice, Poland; 7 Optegra Clinic in Krakow, 30-347 Krakow, Poland; 8 Department of Ophthalmology, Central Clinical Hospital of the Ministry of National Defense, Military Institute of Medicine in Warsaw, 04-141 Warsaw, Poland; 9 Optegra Clinic in Warszawa, 02-366 Warszawa, Poland

**Keywords:** refractive lenticule extraction small incision lenticule extraction, ReLEx, SMILE, cap, residual stromal thickness (RST), refractive surgery

## Abstract

**Background**: In this study, the changes in corneal cap and residual stromal thickness (RST) values during a 180-day observation period after refractive lenticule extraction small incision lenticule extraction (ReLEx SMILE) were assessed. **Methods:** Fifty patients underwent ReLEx SMILE using the VisuMax 500 femtosecond laser, with corneal imaging conducted pre and post procedure via anterior segment optical coherence tomography (AS-OCT). Cap thickness in the center and 1.5 mm from the center in four meridians was measured at various intervals. **Results:** The results showed a significant decrease in cap thickness 180 days post procedure compared to earlier intervals (*p* < 0.05). Similarly, RST decreased gradually and significantly post procedure (*p* < 0.05). Notably, changes in cap thickness within the central 1.5 mm area were more dynamic than RST changes during the 6-month observation period following SMILE. **Conclusions**: The corneal cap thickness measured with swept-source AS-OCT within the central 1.5 mm area underwent more dynamic changes than the residual stromal thickness during the 6-month observation following SMILE.

## 1. Introduction

Many patients with eye defects, including people of all ages, wish to eliminate their visual disorders and stop wearing glasses or contact lenses. At present, laser vision correction (LVC) procedures are considered significantly safer than contact lenses, since the risk of infection during the procedure is lower than while wearing contact lenses for more than a year (0.035% for LVC procedures) [1,2,3]. During the qualifying examination, each patient must be individually considered, given that the selection of an LVC method depends on numerous factors, such as cornea parameters, the severity of the visual defect, the patient’s demands and expectations, the patient’s profession, and other forms of activity (sport, hobby) [4,5].

In 2006, W. Sekundo performed the first femtosecond lenticule extraction (FLEx) procedure, in which he did not use an excimer laser to model the cornea, while the entire procedure was based on the activity of the femtosecond laser [6]. In time, the FLEx procedure was replaced with refractive lenticule extraction small incision lenticule extraction (ReLEx SMILE). In the ReLEx SMILE procedure, the femtosecond laser cuts a micro-lens in the corneal stroma (professionally termed as lenticule), which is then removed towards the outside by a 2–4 mm linear cut in the upper part of the cornea. The thickness of the lenticule depends on the severity of the visual defect [7,8].

The ReLEx SMILE method is considered less invasive than FemtoLASIK in the treatment of myopia and myopic astigmatism. It allows for maintaining a better innervation of the cornea and ensures better parameters of cornea biomechanics [9,10]. In 2016, the ReLEx SMILE procedure gained approval from the Food and Drug Administration (FDA) and is currently available all over the world. As of the writing of this paper, over 2 million such procedures have been performed. The only femtosecond laser designed for the described procedure is the VisuMax laser (Carl Zeiss Meditec AG). Its main advantage is a lesser dependence of the post-surgical results on the conditions (humidity and temperature) than excimer lasers [11,12]. In the first stage of the ReLEX SMILE procedure, a specialized suction–applanation ring is positioned on the laser head. The laser then generates four cuts: one along the posterior surface of the lenticule, another along the edge of the lenticule, a third along the anterior surface of the lenticule, and, finally, a 2–4 mm linear entry cut. Once these femtosecond laser incisions have been completed, the lenticule is not immediately ready for extraction due to the presence of tissue bridges between the incised corneal fragments. In the subsequent stage, separation of the co-planar planes of the lenticule is performed using a blunt separator. The lenticule is then carefully extracted from the cornea utilizing tweezers. It is noteworthy that, in the ReLEX SMILE procedure, Bowman’s membrane remains functionally intact, which contributes positively to the biomechanical properties of the cornea. For instance, starting with an initial corneal thickness of 550 μm and removing 100 μm of tissue, the cornea retains approximately 75% of its strength post procedure. Therefore, throughout the ReLEx SMILE procedure, the residual stromal thickness (RST) remains unaffected by the laser beam, whereas the corneal cap undergoes changes associated with cell swelling [11,12].

Patients with myopia of up to −3.0 DS and astigmatism of 3.0 Dcyl are qualified for the procedure. A condition for being accepted for the procedure is a stable visual defect (refraction change no higher than −0.5 D during the previous year). The youngest age boundary is 18 years of age, and there is no set oldest age boundary, as long as there is no lens opacification which impairs visual acuity. The procedure is a controversial topic with respect to children and youths under the age of 18, but it is possible in the case of children with high anisometropia and the risk of amblyopia [13,14]. Research has shown that the ReLEx SMILE procedure is similar to LASIK in terms of the effectiveness, safety, predictability, and stability of its results [15,16,17,18].

The goal of the present prospective study was to evaluate changes in the thickness of the cap and RST measured in the center and 1.5 mm from the center with anterior segment optical coherence tomography (AS-OCT) in a period of 180 days after the ReLEx SMILE procedure.

## 2. Materials and Methods

### 2.1. Ethics

This study was performed in accordance with the guidelines of the 2013 Declaration of Helsinki on human experimentation. Data confidentiality and patient anonymity were maintained at all times. Patient-identifying information was deleted before the database was analyzed. It is not possible to identify the patients on an individual level, either in this article or in the database. Informed consent was obtained from all the patients. Approval for this study was obtained from the Bioethical Committee operating at the Medical University of Poznań, Poland (no. 952/16, 15 September 2016).

### 2.2. Subjects

In the period between January 2016 and July 2017, 50 people qualified for this study: 37 women (74%) and 13 men (26%) with myopic astigmatism. The average age of the study participants was 31.8 ± 5.6 (22–45) years. The inclusion and exclusion criteria for the study group are presented in Table 1.

### 2.3. Anterior Segment Optical Coherence Tomography

Anterior segment optical coherence tomography (AS-OCT) was used with a DRI OCT apparatus (Triton, Topcon, Warsaw, Poland) to obtain images of the cornea from patients before and after the ReLEx SMILE procedure. One day before the procedure and 7, 60, and 180 days after the procedure, we evaluated the thickness of the RST and cap in the center (peak) and 1.5 mm from the center in four meridians—positions 12, 6, 3, and 9. For each patient, the RST and cap values were determined in duplicate. The difference between the measurements was no greater than 1.2%. Each AS-OCT was performed by the same specialist (D.J.-B.).

**Figure 1:**
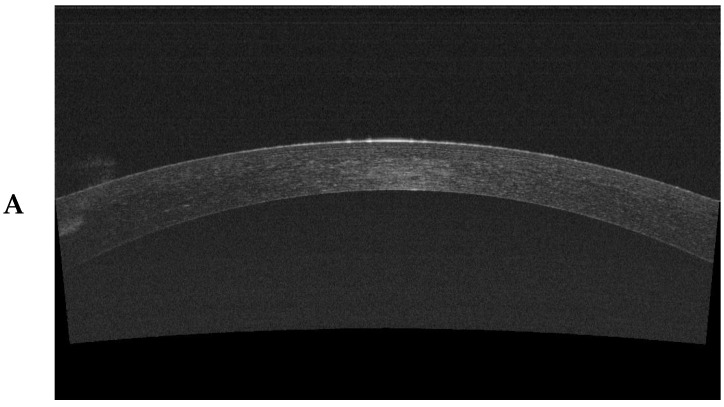

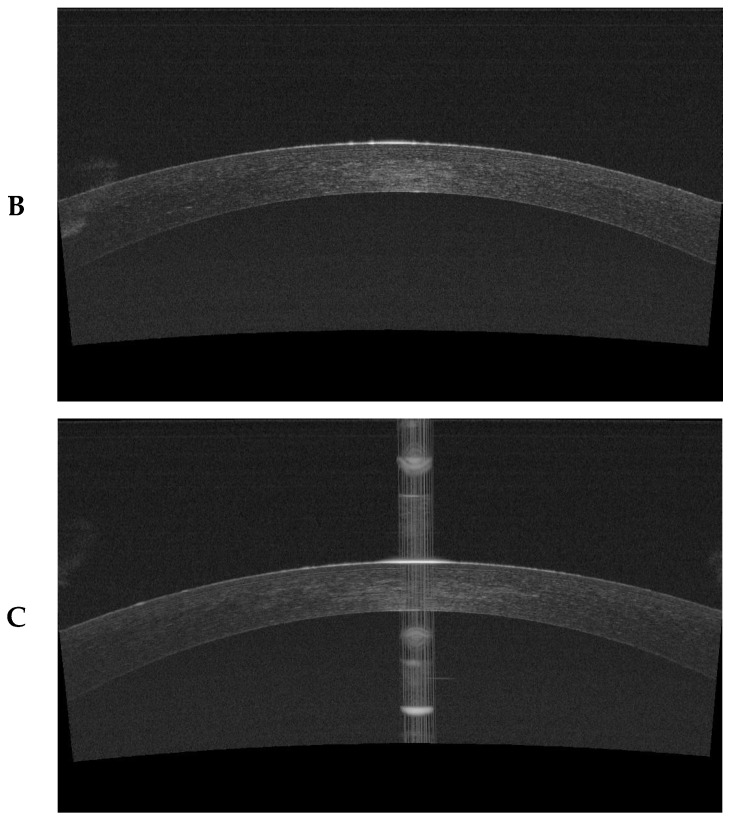
Corneal cross sections were taken with SS-OCT (DRI OCT Triton camera): (**A**) epithelial measurement, (**B**) cap measurement, and (**C**) RST measurement.

The imaging of individual structures was performed to assess the change in corneal morphology during the healing process after SMILE surgery (Figure 1).

### 2.4. Surgical Intervention—The ReLEx SMILE Procedure

The ReLEx SMILE procedure was carried out for all the patients with the use of the VisuMax 500 femtosecond laser (Carl Zeiss Meditec AG, Jena, Germany) at the Optegra Clinic in Poznań, Poland. All the patients were operated on by the same surgeon, with the same laser parameters: energy 140 nJ, spot distance 4.5 m (lenticule and cap), and 2 m (lenticule side and cap side).

After administering local anesthesia to the conjunctival sac with anesthetic drops (Tetracaine Hydrochloride 0.5%, Bausch & LombHouse, Surrey, United Kingdom), ensuring a sterile surgical field, and mounting a stay, an applanation cone coupled with a suction ring was mounted on the laser head. Then, the patient’s head was moved under the laser head. When the patient’s eye was near the ring, the patient was asked to look at the green fixating light placed in the laser head. After ensuring that the patient fixated properly, the ring was placed on the cornea, and a vacuum was created, thus attaching the ring. After the appropriate negative pressure was achieved and eye centration was in line with the optical axis, the femtosecond laser was turned on. The duration of laser activity in each procedure was approximately 30 s. The laser performed the incisions in the following order:

- cutting posterior lamellar plane—refractive
- cutting lenticule side
- cutting anterior lamellar plane—neutral
- cap side cut—port—position 115°

The parameters entered into the laser were as follows: sphere from −1.0 to −10.0 D; cylinder up to −3.5 D; SE from −1.0 D to −10.0 D; lenticule parameters—optical zone (diameter) from 6.3 to 7.0 mm, minimal thickness from 15 to 25 mm, and maximal thickness from 49 to 174 mm; cap parameters—diameter from 7.4 to 7.9 mm, and thickness from 110 to 135 m; entry parameters—length from 3.21 to 3.42 mm, and position 115°; and RST from 251 to 406 m.

After the laser ceased functioning and the negative pressure was removed, mechanical dissection and lenticule extraction were performed by the surgeon. In the first stage, the Sinskey hook (G-33954 Femto Double Instrument, Cresrpoint Ophthalamics, St. Louis, MO, USA) was used to open the entry and find the edge of the lenticule. With the use of the Geuder separator (G-33954 Femto Double Instrument, Cresrpoint Ophthalmics, St. Louis, MO, USA), the upper followed by the lower surface of the lenticule were then dissected. After the complete dissection of the lenticule, the surgeon used the separator to remove the lenticule from the port, to the outside. For every patient, after the lenticule had been extracted, its shape was checked for complete dissection. After the operation, all the patients received antibiotic drops (moxifloxacin 5 mg/mL, Novartis Poland, Warsaw, Poland) 4 times a day for 7 days, steroid drops (loteprednol, 5 mg/g Baush & Lom House, Surrey, United Kingdom) 4 times a day for 14 days, and moisturizing drops (trehalose, hyaluronic acid Thea Laboratories, Warsaw, Poland) 5 times a day for 3 months.

### 2.5. Statistical Analysis

A statistical analysis was conducted with the use of the STATISTICA 13 software (Statsoft, Cracow, Poland) and the SPSS version 17 software, assuming a significance threshold of (*p*) < 0.05. To evaluate the dependencies between the two qualitative variables, the chi-square test was used. We also used the Shapiro–Wilk test to check whether the spread of the presented data corresponded to the normal spread. Given that our data showed a normal spread, to determine whether the changes in the cap and RST were statistically significant, a one-way ANOVA test was conducted. In cases in which the test results indicated the presence of statistically significant changes, a post hoc Tukey’s test was conducted to identify the observation periods between which the changes had occurred. The homogeneity of variance before the ANOVA test was checked with Levene’s test.

## 3. Results

### 3.1. Analysis of Qualitative Changes That Characterized the Study Group

The statistical analysis of the qualitative changes characterizing the study group did not show any relationship between the evaluated variables (Table 2).

### 3.2. Changes in Cap Thickness during the Observation

The regularity of the cap *in the center*, expressed as cap thickness, was statistically significantly lower in the 180-day period after the procedure (*p <* 0.05). Only between day 30 and day 180 of the observation period were the changes in cap thickness in the center not statistically significant (*p >* 0.05). The cap thickness measured 1.5 mm from the center at the four meridians—positions 12, 6, 3, and 9—was statistically significantly lower in the 180-day observation period after the procedure (*p <* 0.05). The changes in cap thickness, along with the results of the post hoc Tukey’s test, are presented in Table 3.

### 3.3. Changes in the RST during the Observation

We noted a gradual but statistically significant decrease in the RST both in the center as well as in the four meridians (*p <* 0.05). The changes in the RST, along with the results of the post hoc Tukey’s test, are shown in Table 4.

## 4. Discussion

Optic coherence tomography of the anterior segment of the eye (AS-OCT) has become an important diagnostic tool for patients prior to and after LVC treatment. Recent studies have focused on evaluating the thickness of the cap and RST after the ReLEx SMILE procedure. Although SMILE is considered a safe, effective, and predictable method in the treatment of myopia and myopic astigmatism, it is important to understand the influence of cap morphology on refractive results. AS-OCT is very useful in analyzing the morphology of the cornea, since it is a contact-free method which is easy to use and allows for the view of a large surface of the cornea at different depths, as well as for the direct measurement of the cap thickness in different meridians [19,20,21,22,23,24].

Zhao et al. [25] demonstrated that the cap created by the femtosecond VisuMax laser is characterized by a high degree of repetitiveness, regularity, and morphological uniformity. In the present study, the cap thickness in the center was lower in the sixth month than on the first day and in the first week, indicating that the cap thickness decreased over time after the surgery. A high cap regularity plays a significant role in maintaining corneal biomechanics and obtaining satisfactory refractive results [25]. The VisuMax laser 500 Hz creates a cap with a high predictability and a uniform morphology, which has been shown in numerous works [21,22]. Reinstein et al. [21] showed that the cap created by the VisuMax laser during the SMILE procedure is characterized by a high accuracy and repetitiveness when it comes to thickness compared to the flap created with the aid of the Hansatome microkeratome in the central 5 mm of the cornea diameter. The standard deviation for the femtosecond laser is 4.3 mm, whereas, for Hansatome, it is 10.7 mm [21]. It is extremely difficult to compare cap and flap thickness in individual studies since the thickness is measured in different locations and with the use of different measuring devices.

In our work, the cap thickness in the center was found to be 144.27 ± 15.85 μm on day 1 after the procedure, 141.20 ± 13.89 µm on day 7, 136.56 ± 12.03 µm on day 60, and 135.05 µm ± 11.22 µm 180 days after surgery. The cap thickness measured in the center was statistically significantly lower 180 days after the surgery compared with the thickness 1 day after the procedure. At a distance of 1.5 mm from the center, in all positions, the cap thickness on day 180 was also statistically significantly lower in comparison with the thickness on day 1. In our study, the cap thickness was higher in the center than the cap thickness 1.5 mm from the center during the entire observation period.

Tay et al. used OCT RTVue (OPTOVUE) to show that the cap on the cornea perimeter was thicker than in the center, and its shape was similar to that of a concave meniscus [23]. In their study, the average cap thickness was statistically higher on the perimeter at distances of 1.5 mm and 3 mm from the center in comparison to the center [23]. In our study, we observed a completely opposite dependency. In Tay et al.’s study, changes in cap thickness were not statistically significant at the observed positions for 6 months. Only in position −3.0 mm in the third month after the surgery was the cap significantly thinner in comparison with the first week and first month and remained the same until the sixth month [23].

Fu et al. studied cap morphology, dividing the cornea into concentric rings with a radius of 1.5 mm (central), 2.0–2.5 mm from the center (para-central), and 3.0–2.5 mm from the center (peripheral) [24]. Using OCT RTVue (OPTOVUE), they showed that, in all areas, the cap thickness statistically significantly decreased only between the first day and first week (*p* < 0.05) [24]. We observed the opposite dependency in our study. There were no significant differences between the first and seventh day after the procedure in the cap thickness in all areas, besides a reduction in the cap thickness in the center, at position 9. Cap thickness significantly decreased in all positions between day 7 and day 180 of the observation period (*p* < 0.05). Fu et al. showed that the cap thickness in the center was significantly lower than the cap thickness in both the paracentral and peripheral areas [24]. By contrast, the cap thickness in the center was the highest in our study.

Zhao et al. evaluated the cap with the use of the OCT RTVue (OPTOVUE) in the center and at distances of 1.5 mm, 2.5 mm, 3.25 mm, and 3.75 mm for the center in meridians 0, 45, 90, and 135 degrees for 54 patients. They did not show any statistically significant changes in cap thickness in the center between the first day and first week after the surgery, as opposed to the results obtained by Fu et al. and those presented in our study. It was not until 1 month and 6 months after the procedure that the cap thickness in the study completed by Zhao et al. was statistically significantly thinner than the thickness 1 day and 1 week after the procedure [25].

In our study, only the central thickness of the cap between the second month, first week, and first day was significantly thinner. There were no statistical changes between the second and sixth month.

Zhao et al., in comparing the cap thickness at individual points on each meridian, noticed a high degree of cap regularity during their observation. However, no significant changes between the central point or points on the perimeter in the four meridians were reported [25]. The authors also showed that cap regularity and thickness play a very important role in ensuring appropriate biomechanical properties, which guarantee satisfactory refractive results. They observed that cap thickness was the greatest 7 days after the surgery and then decreased until the sixth month. This is connected with the postoperative swelling of the cornea, which gradually recedes until the 1st month after the procedure and stabilizes by the 6th month [25], consistent with our observations. In the present work, we did not perform an analysis of the influence of cap morphology on refractive results after the procedure, or of cap thickness between the individual observed points. Thus, further study and observation are needed to elucidate the effects of these factors.

Previous studies have compared cap thickness after SMILE with flap thickness after LASIK, reporting that after the LASIK procedure, the flap becomes thicker (edema) for an extended period of time in comparison with the cap [26,27,28]. Various processes occurring in the early postoperative period may influence the difference between LASIK and SMILE. These include liquid resorption induced by intraoperative irrigation, changes in cornea hydration, and changes in the thickness of the cornea epithelium as a response to the healing process. In these two types of procedures, the healing process and the inflammatory response also have different course [29]. Riau et al. demonstrated that the excimer laser in LASIK stimulates a higher inflammatory response in comparison with the femtosecond laser in ReLEx SMILE [30]. During the SMILE procedure, there was minimal damage to the epithelium and Bowman’s membrane in comparison with LASIK, which resulted in less swelling of the cornea after SMILE. The degree of the inflammatory response also depends on the frequency of the energy supplied to the cornea by the femtosecond laser. Hence, the cap-healing mechanism after the ReLEx SMILE procedure and changes in cap morphology have not yet been fully understood [30,31,32].

The second parameter we evaluated across time was changes in the RST. In the RST analysis, the thickness was lowest in the center. Across all positions, compared to day 1, day 7 and day 180 showed a decrease in the RST in the center and 1.5 mm from the center. However, a comparison of days 7 and 60 showed a significant increase in the RST in the center and in position 3.

Tay et al. observed an increase in the average RST in the center and on the perimeter between the first week and the third month after the procedure, and then the RST remained stable until the sixth month [23]. The average increase in the RST between the first week and the third month was 6.09 ± 2.51 μm. The RST was statistically thinner in the center compared with the RST 1.5 mm and 3 mm from the center after 3 months [23]. Our results contradict those of Tay et al., who reported an increased RST, whereas we observed a decrease over 6 months [23]. The only similarity between our findings and Tay et al.’s is that the RST in the center was lower than the RST 1.5 mm from the center during the entire period of observation [23]. Tay et al. used OCT RTVue (OPTOVUE), which is FD-OCT, whereas we used AS-OCT [23].

In a study on the morphology of the cornea after LASIK, Maldonado et al. noticed a significant increase in the central thickness of the RST in the first and third month after the procedure with the use of Hansatome keratome [33]. Li et al. observed that the RST after LASIK (Hansatome) as well as after FemtoLASIK (IntraLase—Abbott Medical Optics) differed in the first week after the procedure. In the FemtoLASIK group, the RST value decreased, whereas, in the LASIK group, it increased over a period of 3 months [34].

The differences in the RST after the LASIK procedure, both with the use of the femtosecond laser and Hansatome, depend on numerous factors, such as altered biomechanics of the cornea, excessive surgical manipulation, and excessive intraoperative irrigation. According to Tay et al., early changes in RST are more connected to the operation process and its biomechanical effect than to the reconstruction of the proper substance of the cornea, which takes place later [23]. In their study, Riau et al. measured the RST and changes in this parameter after the SMILE procedure; however, their results were inaccurate and inconclusive. In their study, some measurements of the corneal thickness after surgery were higher than the same measurements prior to the procedure [30]. Similar observations were made by Wang et al. [35]. Further, it is very difficult to compare the cap and RST after SMILE to the thickness of the flap and RST after LASIK and FemtoLASIK. There are quite large differences in the thicknesses of the flap and RST after LASIK, which depend on the method used for creating the flap, the duration of the study, and the type of OCT used in the study [23].

Of course, the results we obtained and presented constitute a premise for further research. In the present work, we did not conduct an analysis of the influence of cap morphology on the refractive results after the procedure. In further studies, it would also be reasonable to evaluate the cap and the RST in shorter periods. This would allow for a better evaluation of the dynamics in the changes in the cap and RST after the ReLEx SMILE procedure. Notably, only a few studies have examined the long-term changes in the RST after ReLEx SMILE in OCT. A regression analysis of the cap changes against age or refractive defect size was not performed. In addition, we did not perform a regression analysis of the cap changes against age or the size of the refractive defect, nor did we evaluate concurrent changes in the epithelium during follow-up. In addition, an interesting idea for future studies would be to determine the examination of intracellular edema after ReLeX SMILE using confocal microscopy. Moreover, a potential limitation of our work may be the lack of inclusion of a control (comparison) group in which the vision defect was removed by another method, e.g., like in the work of Bohac et al. [36]. However, when assessing changes in cap thickness and RST, patients undergoing another method of laser vision correction cannot be used as a control (comparison) group. This is due to the fact that, in the case of FemtoLASIK, there is ablation of corneal parenchyma cells and exposure of the stroma, which is not observed during ReLeX SMILE, where no ablation of corneal cells is observed, only intracellular edema, and the stroma is not exposed.

An important factor that limited our work was the relatively short observation time. Since its appearance in refractive surgery, the many advantages of the flapless SMILE procedure compared to the LASIK procedure have been demonstrated numerous times. These advantages include the high stability of refractive results, the maintenance of better cornea biomechanics, the improved resistance of the cap and the external layers of the cornea, the reduction in flap complications, a lower impairment of contrast sensitivity, lower higher-order aberrations of the eye induction, and a weaker dry-eye syndrome.

## 5. Conclusions

The corneal cap thickness measured with AS-OCT within the central 1.5 mm area underwent more dynamic changes than the residual stromal thickness during a 6-month observation following SMILE. The central cap decreased from 1 day to 2 months, whereas the cap thicknesses measured 1.5 mm from the center decreased from 2 month to 6 month after the SMILE procedure. The residual stromal thickness measured within 1.5 mm from the center remained stable after 2 months from the SMILE procedure.

## Figures and Tables

**Table 1:** Inclusion and exclusion criteria for the study group.

Inclusion	Exclusion
Expressing informed consent to participate in this study	Lack of informed consent to participate in this study
Age over 18 years	Younger than 18 years of age
Stable refraction in the year preceding this study	Opaque optical media
Myopia ≤ −3.0 DS	Current or past inflammation of the vascular membrane
Astigmatism ≤ 3.0 Dcyl	Eye defects
CDVA ≥ 0.5 on the Snellen chart	Undergone laser corneal treatment
CT ≥ 490 µm	Undergone surgical treatment of the eyes
RST ≥ 250 µm	Autoimmune diseases
Normal corneal topography	Diabetes
	Pregnancy and breastfeeding

**Table 2:** Analysis of qualitative changes describing the study group.

	Division	Gender		Total	Chi Test Result^2^, *p*-Value
		Female (n = 37)	Male (n = 13)		
**Eye**	Right	18 (49.30%)	7 (52.00%)	25 (50.00%)	Chi^2^ = 0.104 *p* = 0.748
	Left	19 (50.70%)	6 (48.00%)	25 (50.00%)	
**Myopic astigmatism**	No	18 (47.90%)	7 (56.00%)	25 (50.00%)	Chi^2^ = 0.104 *p* = 0.748
	Yes	19 (52.10%)	6 (44.00%)	25 (50.00%)	
**Visual impairment**	No	29 (79.50%)	12 (92.00%)	41 (82.00%)	Chi^2^ = 1.260 *p* = 0.261
	Yes	8 (20.50%)	1 (8.00%)	9 (18.00%)	

**Table 3:** Changes in cap thickness in the first 180 days after the procedure.

	1 Day	7 Days	60 Days	180 Days	*p*-Value
Cap center (µm)	144.27 ± 15.85	141.20 ± 13.89	136.56 ± 12.03	135.05 ± 11.22	0.014 ^1^ <0.001 ^2^ 0.224 ^3^
Cap position 12 (µm)	138.14 ± 11.46	138.68 ± 12.73	134.19 ± 10.21	132.52 ± 8.75	0.598 ^1^ <0.001 ^2^ 0.102 ^3^
Cap position 6 (µm)	143.18 ± 13.31	142.00 ± 12.80	137.54 ± 11.40	132.79 ± 10.58	0.301 ^1^ <0.001 ^2^ <0.001 ^3^
Cap position 3 (µm)	143.57 ± 11.63	142.51 ± 12.70	137.68 ± 10.63	133.81 ± 10.25	0.337 ^1^ <0.001 ^2^ <0.001 ^3^
Cap position 9 (µm)	137.32 ± 12.10	134.96 ± 10.85	132.67 ± 10.55	130.50 ± 8.84	0.024 ^1^ 0.028 ^2^ 0.038 ^3^

mean ± standard deviation; *p*—*p*-value; ^1^, *p*-value in cap thickness between 1 and 7 days after surgery; ^2^, *p*-value in cap thickness between 7 and 60 days after surgery; and ^3^, *p*-value in cap thickness between 60 and 180 days after surgery.

**Table 4:** Changes in the RST in the first 180 days after the procedure.

	1 Day	7 Days	60 Days	180 Days	*p*-Value
RST center (µm)	295.38 ± 41.95	291.80 ± 41.56	294.86 ± 40.2	291.64 ± 40.36	0.012 ^1^ 0.032 ^2^ 0.224 ^3^
RST position 12 (µm)	334.86 ± 37.75	325.33 ± 39.20	326.99 ± 39.32	323.99 ± 39.32	<0.001 ^1^ 0.283 ^2^ 0.052 ^3^
RST position 6 (µm)	335.17 ± 37.55	328.24 ± 39.01	330.05 ± 38.28	329.45 ± 38.67	<0.001 ^1^ 0.184 ^2^ 0.660 ^3^
RST position 3 (µm)	320.80 ± 40.21	315.42 ± 39.05	319.13 ± 38.06	318.80 ± 39.85	<0.001^1^ <0.01 ^2^ <0.816 ^3^
RST position 9 (µm)	334.44 ± 38.02	330.99 ± 39.72	330.29 ± 39.75	327.82 ± 40.54	0.001 ^1^ 0.607 ^2^ 0.072 ^3^

mean ± standard deviation; *p*—*p*-value; ^1^, *p*-value in cap thickness between 1 and 7 days after surgery; ^2^, *p*-value in cap thickness between 7 and 60 days after surgery; and ^3^, *p*-value in cap thickness between 60 and 180 days after surgery.

## Data Availability

The data used to support the findings of this study are included within the article.
